# An integrative, holistic treatment approach for veterans with chronic traumatic brain injury and associated comorbidities: case report

**DOI:** 10.3389/fpsyt.2025.1568876

**Published:** 2025-05-02

**Authors:** Rebekka Dieterich-Hartwell, Janine Brodovsky, Kathryne DeAlba, Allison Rubin, Emily McGuigan, Bindal M. Mehmel, Yevgeniya Sergeyenko

**Affiliations:** ^1^ MossRehab Institute for Brain Health, Jefferson Moss Magee Rehabilitation Hospital, Philadelphia, PA, United States; ^2^ Department of Rehabilitation Medicine, Sidney Kimmel Medical College, Thomas Jefferson University Hospital, Philadelphia, PA, United States

**Keywords:** interdisciplinary, traumatic brain injury, veterans, case report, posttraumatic stress, intensive outpatient program, rehabilitation

## Abstract

This case report of a veteran with a history of multiple traumatic brain injuries, posttraumatic stress, and chronic pain highlights the MossRehab Institute for Brain Health (MRIBH) model of care: a collaborative, interdisciplinary, intensive outpatient treatment program. Unique to this case was the severity of symptomatology and the concerted engagement by the clinicians, who fully tailored and adjusted their approaches based on team rounds and daily exchanges. The client’s main concerns of headaches, difficulty sleeping, difficulty with memory and cognition, depression, anxiety, posttraumatic stress, anger, and chronic musculoskeletal pain were addressed and significantly improved within three weeks. The outcome of this case is promising and suggests that the highly individualized and collaborative interdisciplinary approach provided at MRIBH is particularly beneficial for veterans with chronic TBI and PTSD and can provide a roadmap for the treatment of specific clinical scenarios.

## Introduction

Traumatic brain injury (TBI) is the signature injury of the recent military conflicts in Afghanistan and Iraq [Operation Enduring Freedom (OEF)/Operation Iraqi Freedom (OIF)] (1). The Department of Defense (DOD) TBI Center of Excellence (TBICoE) estimates that 514,583 service members (SMs) have been affected by TBI since 2000 (2). TBI can result in physical, cognitive, and emotional symptoms, which can last for months to years after the initial injury. In addition to TBI, many SMs also have co-occurring mental health diagnoses, sleep disorders, and chronic pain. Among OEF/OIF veterans with a diagnosis of TBI using Veterans Health Administration services in 2009, 89% of those with a TBI diagnosis were also diagnosed with a psychiatric diagnosis, the most common of which was posttraumatic stress disorder (PTSD) at 73% (3). Similarly, 70% of OEF/OIF veterans had a diagnosis of head, back, or neck pain (3). Co-occurring mental health diagnoses and chronic pain complicate the treatment of TBI in veterans as the wide-ranging symptoms of TBI, PTSD, and chronic pain can overlap and interact to perpetuate and exacerbate one another, leading to decreased function and quality of life. There is mounting evidence of the efficacy of intensive outpatient programs (4–9) in the treatment of SMs and veterans with TBI and co-occurring mental health conditions. However, much of the research is focused on SMs and veterans with mild TBI, and it is unclear if this model will be similarly effective in the treatment of SMs and veterans with moderate-to-severe TBI.

MossRehab Institute for Brain Health (MRIBH) is an innovative, Philadelphia-based, intensive outpatient program (IOP) that provides interdisciplinary treatment for veterans and first responders (FRs) experiencing persistent symptoms of TBI and co-occurring mental health conditions, at no out-of-pocket cost. This philanthropically funded program serves patients from across the country. The clinical team at MRIBH consists of co-located specialists from multiple disciplines, including Behavioral Health (BH), Physical Medicine and Rehabilitation/Brain Injury Medicine (PM&R), Physical Therapy (PT), Occupational Therapy (OT), Speech Language Pathology (SLP), Art Therapy (AT), Case Management (CM), and Neuropsychology (NP). During the 3-week IOP, veterans and FRs undergo treatment in cohorts of up to five patients, including a combination of individual and group treatment with each of the disciplines, totaling approximately 90 hours of treatment over three weeks. More specifically, patients attend three 60-minute individual sessions per week in each discipline. During the 3-week IOP, patients also participate in three BH groups co-facilitated with AT (Emotional Regulation, Sleep 101), one to three AT groups, three implementation groups (co-facilitated by OT and SLP) with outings into the community (grocery shopping and cooking a meal together, bowling or mall visit), a K-9 group (with the OT and facility dog), a 2-hour CBT-based pain management education group (Empowered Relief®), four movement groups (two led by PT, two focused on dance/movement therapy with BH, two of each co-facilitated by OT), a TBI 101 group (PM&R or NP led), three horticultural therapy groups, and four trauma-informed yoga groups. Unique to MRIBH are the many interdisciplinary, co-facilitated groups, as well as the multiple specialties each clinician brings to the program. For example, the OT provides animal-assisted therapy and remedial vision rehabilitation and also leads the Empowered Relief® pain group; the PT is a certified therapeutic pain specialist and is also certified in vestibular therapy; and the BH specialist is a board-certified dance/movement therapist and is also trained in multiple trauma-informed treatment modalities, such as EMDR (eye-movement desensitization and reprocessing) and hypnotherapy. In addition to the co-facilitation, the clinical team meets weekly in a formal, hour-long team rounds, in which each patient’s treatment goals and progress are discussed by the treatment team. This allows for multiple clinicians to work with the patient on addressing a specific goal and for carry-over of strategies from one specialty to another, increasing the strength of the interventions. Another innovative aspect is the supported immersion into the community and the small, cohort-based model that invites socialization and informal practice of tools on the weekends.

In this report, we present the case of a veteran with a history of multiple TBIs, including a moderate to severe TBI, who successfully completed treatment in the 3-week IOP and had significant improvement in his symptoms. Through our discussion of the case, we illustrate how the team at MRIBH employs a tailored, patient-centered, interdisciplinary approach during evaluation and treatment. Unique to this case is the severity of the patient’s TBI, as well as the severity of his co-occurring symptoms.

## Patient information and clinical findings

Mr. Doe is a 47-year-old Army and Navy veteran who self-referred for evaluation at MRIBH. He endorsed a history of multiple TBIs; his most significant TBI occurred in 2017 secondary to a motor vehicle accident. The car he was driving collided with a semi-truck, and he sustained multiple injuries, including a moderate to severe TBI (right frontal intraparenchymal hemorrhage, possible diffuse axonal injury, cerebral edema); open basal skull fracture with extension into the carotid canal, temporal bone, and right vertebral foramen; multiple facial fractures, including bilateral orbital wall fractures, bilateral maxillary sinus fractures, right mandibular fracture, nasal septum and pterygoid fractures; 1st rib fracture; mild anterior wedge deformities at C5-C6; bilateral lung contusions, right index finger extensor tendon injury; right hand laceration; and multiple facial lacerations. He was intubated for airway protection on arrival to the acute care hospital and underwent surgical repair of his facial fractures, right finger extensor tendon injury, and facial lacerations. He was hospitalized for a total of 10 days and was ultimately weaned off the ventilator and extubated. Acute care hospital course was complicated by agitation, requiring behavioral medications and restraints. He was weaned off behavioral medications prior to discharge. At the time of discharge, he was documented to be awake, alert, and oriented x4; tolerating an oral diet; voiding; and ambulating. He was discharged home with his father.

**Figure 1:**
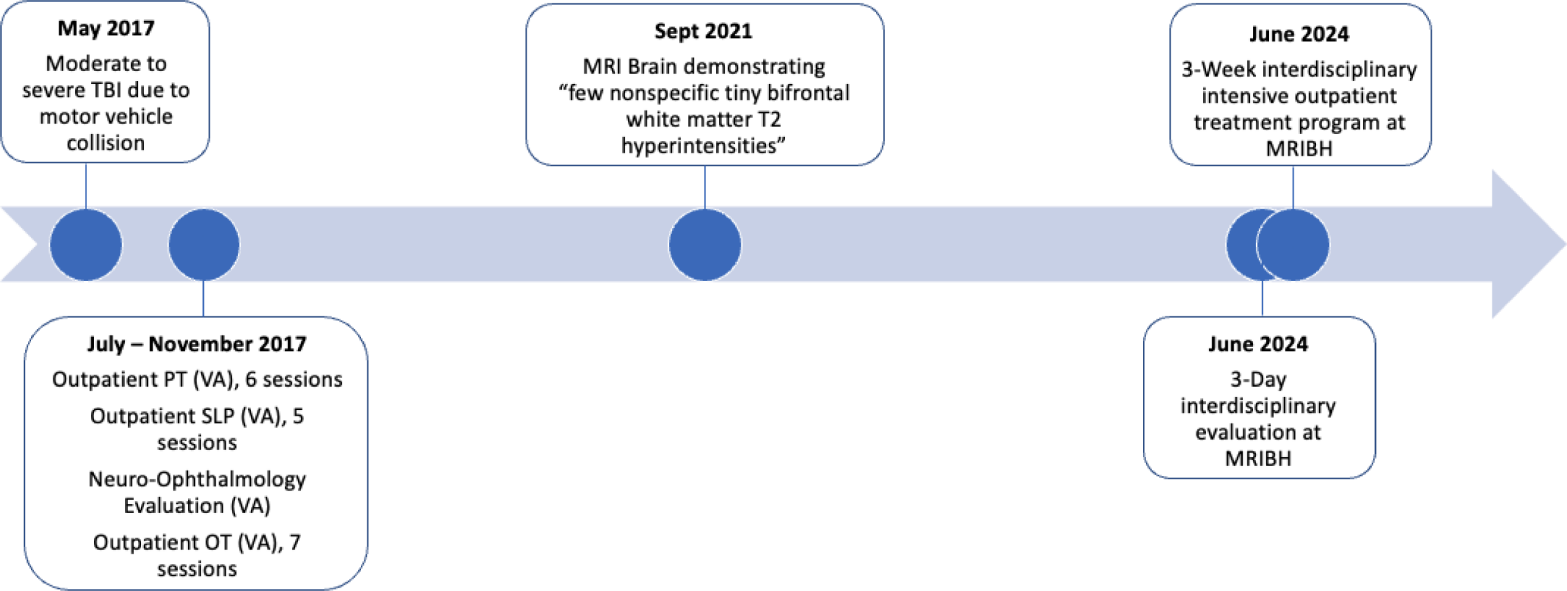
Timeline. Therapeutic interventions.

Mr. Doe did not have any inpatient rehabilitation treatment. Prior to his TBI, Mr. Doe had been working full-time in IT (Information Technology). He was unable to return to work after his TBI. Mr. Doe followed up at his local VA approximately two months after his TBI and at that time, was referred to SLP for outpatient cognitive rehabilitation (5 sessions), Neuro-ophthalmology, and vestibular PT for ongoing dizziness and dysequilibrium (6 sessions). He was additionally referred to OT for ongoing hand pain, neck and upper back pain, and significant binocular vision dysfunction (7 sessions). **Figure 1** depicts a timeline of the therapeutic interventions.

Prior to evaluation at MRIBH, Mr. Doe received regular diagnostic workup and treatment at his local VA, including laboratory studies and MRI brain and cervical spine without contrast in September 2021. MRI brain demonstrated “few nonspecific tiny bifrontal white matter T2 hyperintensities” and MRI cervical spine was notable for “mild spinal canal stenosis at C6/C7 and moderate right neural foraminal stenosis at C6/C7.” For his headaches and migraines, Mr. Doe had previously been treated with botulinum toxin injections (had worsening of his headaches with first round of injections, did not pursue another round) and triptans (found this helpful, but forgot to ask for a refill and was not prescribed triptans at the time of his evaluation). For his mood, Mr. Doe was seeing a psychiatric nurse practitioner at his local VA for medication management. He was on duloxetine at the time of MRIBH evaluation (did not feel this was helping) and had previously trialed sertraline and buspirone. He was also receiving monthly counseling at his local VA.

Despite these interventions, at the time of his evaluation at MRIBH in 2024, Mr. Doe reported his main concerns included chronic daily headaches; severe migraines, which occurred at least twice per week; insomnia; problems with attention, memory, processing, and executive function; severe depression with frequent passive suicidal ideations; post-traumatic stress; impaired balance; and chronic musculoskeletal pain. On physical examination at the time of his evaluation, Mr. Doe was ambulating with a single point cane. He was noted to have tenderness to palpation over his bilateral greater and lesser occipital nerves, auriculotemporal nerves, supratrochlear nerves, and supraorbital nerves. Active range of motion of neck and lumbar spine were limited by pain in all planes, with tenderness to palpation over the spinous processes of the lower cervical and thoracic vertebrae. There was paraspinal muscle tenderness diffusely in the cervical and thoracic spine.

### Diagnostic assessment

During the 3-day evaluation, Mr. Doe was assessed by each clinician using discipline-specific evaluation measures. Time spent with each clinician and clinical findings, including limitations and difficulties in all areas are summarized in **Table 1**.

**Table 1:** Diagnostic assessment and findings.

Discipline-specific Evaluation Measures	Clinical Findings Informing Treatment
Physical Medicine and Rehabilitation (120 min)	
History of present illness, including review of head injury history, current symptoms, and medications. Focused head and neck examination. Neurological examination, including cognitive screening and examination of balance, coordination, vestibular ocular function. Musculoskeletal examination, with a focus on spine.	Tenderness to palpation over bilateral greater and lesser occipital nerves, auriculotemporal nerves, supratrochlear nerves, and supraorbital nerves. Active range of motion of neck and lumbar spine limited by pain in all planes, with tenderness to palpation over the spinous processes of the lower cervical and thoracic vertebrae. Paraspinal muscle tenderness diffusely in the cervical and thoracic spine.
Neuropsychology (240 min)	
Test of Premorbid Functioning, Wechsler Adult Intelligence Scale –4th edition (WAIS-IV-select subtests including Digit Span and Similarities), Repeatable Battery for the Assessment of Neuropsychological Status (RBANS), Trail Making Test, Wisconsin Card Sorting Test (WCST), Conners Continuous Performance Test-3rd edition (CPT-3), Controlled Oral Word Association (COWAT), Personality Assessment Inventory (PAI).	Cognitive: Difficulties with attention, specifically inattentiveness and vigilance; verbal, auditory, and visual learning and memory; cognitive flexibility and set shifting; and categorical fluency. Below expected performance in processing speed and visuoconstruction. Emotional: symptoms of somatization, posttraumatic stress, anxiety, depression, affective instability, aggression, hypervigilance, suicidality, social detachment, stress, and nonsupport
Physical Therapy (120 min)	
Functional Gait Assessment (FGA), Activities of Balance Confidence (ABC), Dizziness Handicap Inventory (DHI), Mean Gait Speed (MGS), Vertigo Symptom Scale (VSS) Patient Specific Functional Scale (PSFS), Pain Rating Scale (PRS).	Imbalance, dizziness, slowed gait speed, poor tolerance for busy visual environments, limited activity pattern due to decreased endurance and headache, neck and back pain.
Occupational Therapy (120 min)	
Canadian Occupational Performance Measure (COPM), OculoMotor Assessment Tool (OMAT), Brain Injury Vision Symptom Survey (BIVSS), Worth 4- Test, Modified Thorington Test, Stereo Fly Test, Pillbox Test, 9-hole peg Test, Hand Dynamometry, Adult Sensory Profile.	Limited activity pattern, poor hygiene, impaired convergence, pursuits, and vertical/horizontal saccades, impaired bilateral upper extremity coordination, decreased bilateral grip strength, decreased attention to detail, high scores in low registration, sensory sensitivity, and sensation avoiding.
Speech Language Pathology (120 min)	
Hearing Handicap Inventory Screening Questionnaire for Adults (HHI), Pure tone hearing screening, Reflux Severity Index (RSI), Eating Assessment Tool (EAT-10), Voice Handicap Index (VHI-10), Neuro-QOL Speech Difficulties self-assessment, Newcastle Dysarthria Assessment Tool, La Trobe Communication Questionnaire, Brisbane Evidence Based Language Test (ELBT), Neuro-QOL Cognitive Function self-assessment, Ross Information Processing Assessment (RIPA-2) (first subtest-immediate recall), Functional Assessment of Verbal Reasoning and Executive Strategies (FAVRES) (1st & 2nd subtest "planning an event" and "scheduling task").	Intermittent slurred speech, tangential speech, word finding difficulty, decreased accuracy following directions, reduced reading comprehension, decreased attention to detail, impaired immediate & working memory, impaired delayed recall, and impaired executive functioning skills.
Behavioral Health Therapy (60 min)	
PHQ-9 (Patient Health Questionnaire), PCL-5 (PTSD checklist for DSM5), GAD-7 (Generalized Anxiety Disorder), Moral Injury Scale, Dissociation Symptoms Scale, Obsessive Compulsive Inventory, ACE (Adverse Childhood Experiences), Insomnia Severity Index.	Severe depression, passive suicidal ideation, severe anxiety, PTSD (elevations across all domains), moderately severe insomnia, moderate moral injury, daily dissociation (particularly cognitive reexperiencing), 6 ACEs, self-blame, ruminating, negative outlook on life.
Art Therapy (60 min)	
The tree assessment creates a dialogue about patient self-perception and perception of growth. Each part of the tree can represent a different part of the patient, e.g. the roots represent background/upbringing or grounding ability. The overall image can suggest external or internal states.	The roots of the tree were a woman floating in a pool, the trunk was a man with his hand over his heart, and the branches were a lighthouse, a rod of Asclepius, a sailboat and a ship. A main focus was the root system being based on his mother, explaining she has consistently been his support.

### Therapeutic interventions

Following the interdisciplinary evaluation, Mr. Doe was recommended to undergo treatment in the 3-week IOP. Mr. Doe began treatment in the IOP the following week. During the 3-week IOP, he received treatment from multiple disciplines including PM&R, PT, OT, SLP, Integrative Therapies, AT, BH, dance/movement therapy and CM (see description of program above). He was part of a 4-patient cohort.

Based on the assessment findings, the same clinicians who evaluated Mr. Doe developed a client-specific plan for the 3-week IOP. Clinicians collaborated via weekly team meetings, a secure healthcare collaboration platform (TigerConnect), and frequent informal exchanges to adjust and complement their approaches throughout the duration of treatment. Team meetings lasted one hour (approximately 15 minutes per patient) each week and detailed the patient’s treatment goals and progress, as well as each clinician’s session contents and clinical impressions. As mentioned above, multiple sessions were co-facilitated and interdisciplinary.

### Physical medicine and rehabilitation and brain injury medicine

Mr. Doe was seen for medical follow-up weekly during the 3-week IOP. The focus of medical treatment was management of headaches and sleep. For headaches, Mr. Doe was started on propranolol in the first week of the IOP. Unfortunately, he experienced worsening dizziness with this medication, so it was discontinued. Additionally, Mr. Doe underwent peripheral nerve blocks of his bilateral greater occipital nerves, lesser occipital nerves, auriculotemporal nerves, supratrochlear nerves, and supraorbital nerves, as well as trigger point injections in his neck. To address both headaches and sleep, Mr. Doe was recommended to increase his amitriptyline dose from 25mg nightly to 50mg nightly, and he did notice some improvement in his headaches and sleep with this. To address any potential visual contribution to his headaches, Mr. Doe was referred to neuro-optometry for evaluation.

### Physical therapy

Mr. Doe participated in PT targeting dizziness, imbalance, motion sensitivity, pain, slowed gait speed, and decreased activity tolerance. Treatment focused on promoting gaze stability with head movements, balance control, motion habituation and pain neuroscience education. Gait training and balance in the clinic was performed during the first week. He was encouraged to begin daily walks outside of session to increase his endurance. Gaze exercises were initiated in week 2, along with immersive virtual reality training to develop balance strategies, reduce his motion sensitivity in busy visual environments (e.g., the supermarket) and decrease his movement avoidance. Feedback from scoring on different virtual reality modules helped him to see his progress and carry it over into his daily activities. Intensity and duration of treatment was increased during week 3, and Mr. Doe received updated home programs for aerobic conditioning, balance and gaze stabilization. Pain neuroscience education was provided to give him an understanding of why he is experiencing pain and how to modulate it with exercise, breathing, yoga, and meditation.

### Occupational therapy

Mr. Doe participated in OT targeting remedial vision rehabilitation, fine motor coordination, grip/pinch strengthening, canine assisted therapy, functional cognition, and participation in activities of daily living (ADLs) and instrumental activities of daily living (IADLs). Treatment initially focused on establishing good habits and routines, performing meal planning and preparation consistently, and increasing his independence with medication management. Mr. Doe was seen by a neuro-optometrist during the first week of the IOP; he subsequently participated in binocular and oculomotor training in OT to address his diagnosis of convergence insufficiency and oculomotor dysfunction. He was given a comprehensive vision home exercise program including the HTS2 vision computer program, prescribed by the neuro-optometrist. Mr. Doe also received a home program to address his bilateral upper extremity grip/pinch strength and coordination. He participated in canine assisted therapy with a Canine Companions facility dog to address his functional cognition, activity tolerance, and fine motor skills. Mr. Doe implemented cognitive, visual, and sensory strategies during interdisciplinary group community outings (led by OT and SLP) to the grocery store (first week) and bowling alley (second week). He responded well to the treatment sessions and established a plan to carry over the strategies learned when discharged to his home environment.

### Speech language pathology

Mr. Doe participated in skilled speech therapy treatment targeting speech production, pragmatic skills, word finding, reading comprehension, attention, memory, and executive functioning skills. Therapy was based off standardized test results, self-assessments, and clinical observations (detailed in **Table 1**). The focus of the sessions included education on areas of weakness identified in the speech therapy and neuropsychological evaluations, training on the use of compensatory strategies, and practice using strategies to improve functioning. Mr. Doe reported feeling self-conscious about his speech given history of facial trauma resulting in a scar on the inside of his lip, so he was given articulation exercises to complete as homework. Education on areas of weakness and training on the use of compensatory strategies occurred during the first week of IOP. During the second and third weeks of IOP, Mr. Doe worked on utilizing strategies, such as semantic feature analysis, to assist with word finding difficulty; intentional reading and preview, question, read, summarize, test (PQRST) to assist with reading comprehension; internal memory strategies, such as association and visual imagery, to help with recall; and divide and conquer to help with completing overwhelming tasks. Mr. Doe responded well to treatment and exhibited carry-over of strategies outside of the treatment room. He began implementing strategies learned in other therapies (e.g., BH, AT), such as reframing his thinking to be more positive in SLP.

### Behavioral health therapy

Mr. Doe’s main BH goals were to increase emotional regulation and self-love and address a specific traumatic incident. Both therapist and Mr. Doe decided together that EMDR therapy would be a good modality to try as it can be effective in short-term treatment, especially when there is an isolated traumatic experience. The sessions in the first week focused on grounding and psychologically managing his severe headaches, as well as a calm place exercise with bilateral stimulation (butterfly tapping). Mr. Doe identified “pure relaxation” and tapped into his sensations and breath (particularly sighing) to help him relax and release tension. He also resonated with the sentiment of being nonjudgmental but compassionate towards himself. In the second week and the beginning of the third week, Mr. Doe reprocessed the traumatic experience of his accident through EMDR. While recalling this memory, Mr. Doe initially identified a great deal of anger and sadness but gradually made peace with himself and this experience. He recognized that there was purpose to him and his story. Challenging his habitual negative self-talk throughout the third week, and with the specific tools provided during the emotional regulation BH group sessions, he began to express hopefulness with the phrase “I am an overcomer.”

### Dance/movement therapy

Mr. Doe participated in two DMT groups (second and third weeks) and had several associations with the movements, including flying. During the raising and lowering of a parachute, he expressed feeling a unity with his peers, as if they “had known each other for a long time”. When asked to create a movement phrase that symbolized what he was taking from the program, he stated “happiness” and made a body wave that he passed on to the others.

### Art therapy

**Figure 2:**
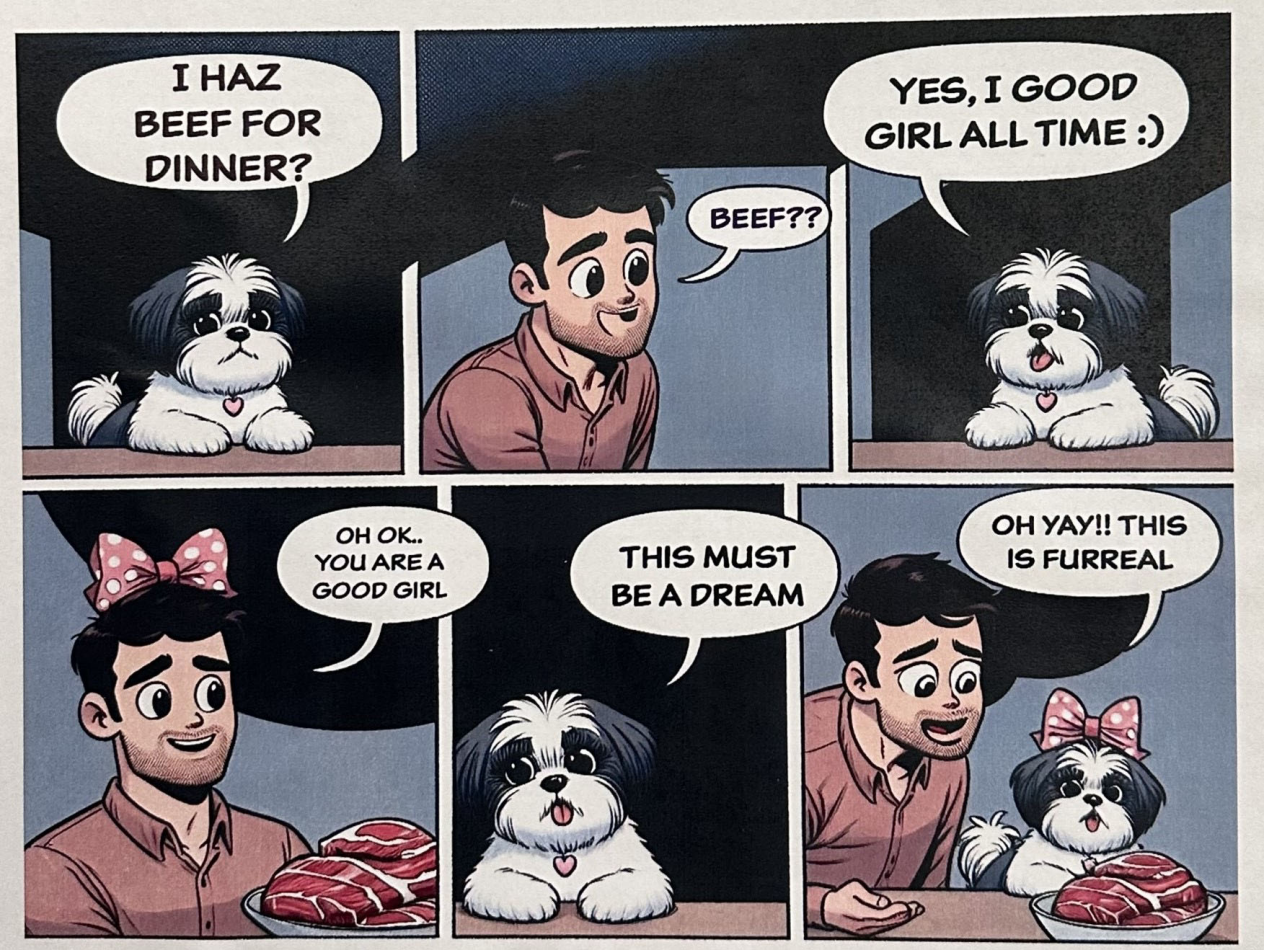
Art Therapy project created by Mr. Doe during week 1. Artificial intelligence (AI) -generated comic strip representing his relationship with his dog.

In AT, Mr. Doe was challenged with recognizing areas of negative thought patterns and how his pain interferes with his daily life/self-efficacy through creative expression. Mr. Doe explored incorporating AI (artificial intelligence) art into his storytelling abilities, and he also created a mask and mosaic. One project he created through AI manipulation during week one was a comic strip representing his relationship with his service dog (see **Figure 2**). Mr. Doe had previously enjoyed creating AI art and demonstrated choice and control by bringing this medium into sessions. He expressed confidence in his ability to navigate the application functions. The therapist facilitated his brainstorming process to see what aspect he wanted to focus on in this new storyline, then he used keywords to generate graphics to match. He was also able to change the verbiage generated. He chose to focus on his service dog requesting a beef dinner in this comic, utilizing his humor. Once finished during the beginning of week two, this comic strip created a conversation centering around how his ability to care for her motivates him to care for himself, which showed carryover from OT sessions focusing on hygiene and ADLs. Mr. Doe’s final project in week three was to create an “I Am” collage to honor himself and the progress he made with his positive self-talk. The prompt was to finish the sentence “I am…” through the use of collage materials. Mr. Doe's self-efficacy greatly improved, as evidenced by his choice of words.

### Follow-up and outcomes

Mr. Doe made great progress with all disciplines. Medically, he reported a subjective “10% decrease” in the severity of his headaches after peripheral nerve blocks, stating that the nerve blocks “turned down the volume of the pain enough that [he was] able to focus on other things.” On the Neurobehavioral Symptom Inventory (NSI), Mr. Doe’s headaches decreased from “very severe” at the beginning of IOP to “mild” at the end of IOP, a difference of 3 out of 5 points. More generally, Mr. Doe’s symptom burden decreased significantly after three weeks of IOP treatment, as reflected in a decrease of 42 points on his Neurobehavioral Symptom Inventory.

In PT, pain neuroscience education gave Mr. Doe an understanding of why he is experiencing pain and how to modulate it with exercise, breathing, yoga and meditation. This knowledge allowed Mr. Doe to understand that he can be active without increasing his pain or injuring himself. Reduction in his dizziness and pain enabled Mr. Doe to increase his activity, balance confidence, exercise tolerance and overall balance performance. This was reflected by significant improvement in balance measures – Activities of Balance Confidence Scale score improved from 35%, indicating low level of physical functioning, to 72%, indicating moderate level of physical functioning and decreased fall risk; Vertigo Symptom Scale score decreased from 34% to 13%, indicating significant reduction in visual vertigo symptoms in busy environments; and Dizziness Handicap Inventory decreased from 78%, indicating severe perception of handicap due to dizziness, to 20%, indication mild perception of handicap due to dizziness. Mr. Doe also had improvement in his mean gait speed (from 2.9 feet per second to 5.29 feet per second, indicating functional gait speed post-IOP) and in his Functional Gait Assessment (score increased from 19/30 to 28/30, reflecting reduced falls risk post-IOP).

In OT, Mr. Doe gradually increased his independence with his ADLs and IADLs throughout the course of the IOP. This included increasing his independence with medication management, hot meal preparation and clean-up, maintenance of a consistent self-care routine, and engagement in community outings. Mr. Doe reported a significant decrease in his vision symptoms and his binocular and oculomotor function improved, as reflected by his ability to tolerate and perform vertical and horizontal saccades on the OculoMotor Assessment Tool. Additionally, his near point of convergence break improved from 14 cm to 4 cm, with the post-IOP score reflecting a normal near point of convergence break.

In SLP, Mr. Doe made the most improvements in his speech clarity, social communication skills, attention, and executive functioning skills (reasoning, planning, organizing). Throughout the program, Mr. Doe began implementing daily schedules on the weekend to establish a routine and improve initiation. Mr. Doe exhibited good insight into deficits expressing that he realized he goes through activities too quickly, which leads to decreased attention to detail and mistakes. He implemented speech strategies, such as talking slower and use of over articulation to reduce slurring, leading to improved speech intelligibility per self-report.

In BH, Mr. Doe improved greatly over the course of three weeks, becoming visibly lighter, more positive, initiative in groups, and compassionate towards himself. The distress level (SUD – subjective units of distress scale) of his traumatic incident dropped from 10 to 0, and he was observed actively using his emotional regulation tools. His depression and anxiety decreased significantly (from severe to mild depression and from severe to minimal anxiety), his posttraumatic stress symptoms improved drastically, and his pain self-efficacy increased from a low to a high level of self-management.

In AT, Mr. Doe made notable improvements, especially with his self-expression, understanding of the mind-body connection, self-compassion and overall mood through the use of creative expression and verbal endorsement.

More detailed and numerical changes in pre-IOP and post-IOP measures can be found in **Table 2**.

**Table 2:** Pre- and post iop discipline-specific measures.

Outcome Measure	Initial	Discharge	Scoring/Details
Neurobehavioral Symptom Inventory (NSI)	76	34	Maximum score 80; higher scores indicate increased symptom severity
Physical Therapy			
Pain: C-spine T-spine	5/10 2/10	0/10 0/10	0 = No pain 10 = severe pain
Mean Gait Speed	2.9 feet / sec	5.29 feet / sec	Functional gait speed
Activities of Balance Confidence Scale Score	35%	72%	50-80% = moderate level physical functioning < 50% = low level of physical functioning <67% = older adults at risk for falls; predictive of future falls
Functional Gait Assessment	19/30	28/30	Falls risk cut-off score = 22/30 Reduced falls risk.
Patient Specific Functional Scale	0%	71%	Yoga 2 times/week, Walk 30 mins daily, Standing 10 mins without increased low back pain
Vertigo Symptom Scale (VSS)	34% = (+) Visual Vertigo	13%	Cut-off is ≤ 12% Significant reduction in visual vertigo symptoms in busy environments
Dizziness Handicap Inventory (DHI)	78%	20%	0-39% = Mild perception of handicap due to dizziness 40-69% = Moderate perception 70-100% = Severe perception
Occupational Therapy			
Canadian Occupational Performance Measure (COPM)	Performance Score: 3/10 Satisfaction Score: 2.5/10	Change in performance score: +6 points Change in satisfaction score: +7.5 points	2 points = minimal clinically important difference
Brain Injury Vision Symptom Survey (BIVSS)	102	17	Normal is less than 31 points
OculoMotor Assessment Tool (OMAT): Near Point of Convergence Break	14 cm	4 cm	Normal is 5 cm or less
OculoMotor Assessment Tool (OMAT): Vergence Eye Movements	Unable to perform	1st 30 sec: 29 2nd 30 sec: 30 Total: 59	Normal is at least 60 vergence eye movements per minute
OculoMotor Assessment Tool (OMAT): Horizontal Saccades	1st 30 sec: 30 2nd 30 sec: 26 Total: 56	1st 30 sec: 64 2nd 30 sec: 49 Total: 113	Normal is at least 106 eye movements per minute
OculoMotor Assessment Tool (OMAT): Vertical Saccades	1st 30 sec: 27 2nd 30 sec: 31 Total: 58	1st 30 sec: 52 2nd 30 sec: 44 Total: 96	Normal is at least 105 eye movements per minute
Hand Dynamometry (Grip Strength Measured in Position 2)	Left Avg: 77 lbs Right Avg: 89 lbs	Left Avg: 85 lbs Right Avg: 81 lbs	Mean for Male, Age 45-49: Left Hand Grip: 100.8 lbs ± SD: 22.8 Right Hand Grip: 109.9 lbs ± SD: 23.0
9-Hole Peg Test	Right hand: 57 sec. Left hand: 48 sec.	Right hand: 42 sec. Left hand: 44 sec.	Mean for Male, Age 45-49: Right hand: 18.8 sec. ± SD: 2.3 Left Hand: 20.4 sec. ± SD: 2.9
Pillbox Test	2 errors	0 errors	A straight pass/fail designation is determined by three or more total errors of any type on the task.
Speech Language Pathology			
Hearing Handicap Inventory Screening Questionnaire for Adults (HHI)	6	4	Max. score 40; the higher the score, the more probability of hearing impairment
Reflux Severity Index (RSI)	28	1	Max. score 45; the higher the score, the more probability of significant reflux disease
Eating Assessment Tool (EAT-10)	10	2	Max. score 40; the higher the score, the more perceived swallowing difficulty
Voice Handicap Index (VHI-10)	4	2	Max. score 40; the higher the score, the more perceived voice deficit
Neuro-QOL Speech Difficulties self-assessment	76	34	Max. score 135; the higher the score, the more perceived speech deficit
La Trobe Communication Questionnaire	84	50	Max. score 120; the higher the score, the more perceived communication difficulty
Neuro-QOL Cognitive Function self-assessment	47	89	Max. score 140; the lower the score, the more perceived cognitive difficulty
Behavioral Health Therapy			
Patient Depression Questionnaire-9 (PHQ-9)	27	7	0-4 minimal; 5-9 mild; 10-14 moderate; 15-19 moderately severe; 20+ severe depression
Generalized Anxiety Disorder-7 (GAD-7)	21	4	0-4 minimal; 5-9 mild; 10-14 moderate; 15+ severe anxiety
PTSD Checklist for DSM-5 (PCL-5)	77	15	Max. score 80; PCL-5 cutoff score between 31-33 is indicative of probable PTSD
Pain Self-Efficacy Questionnaire (PSEQ)	11	47	Max. score 60; > 40 indicates high levels of self-efficacy for managing pain < 30 indicates low self-efficacy for managing pain

## Discussion

Mr. Doe greatly benefited from the combination of clinical interventions over the course of three weeks and improved significantly in all areas of initial concern. Importantly, the clinicians continuously collaborated and communicated with each other to optimize the sessions, take into consideration temporary increases in symptoms such as headaches, and to capitalize on momentum. For example, the positive imagery that surfaced through EMDR resourcing in BH (a peaceful nature walk with chickens and other animals, water, butterflies) also emerged in AT (rainbow mosaic, collage with water images) and strengthened and concretized this resource. He told his story through both words and art and expressed his desire to write his memoir. Both PT and DMT invited Mr. Doe to move more, become more confident with balance, and connect to his body in a positive way. In SLP, he created a to-do-list, which helped him organize and complete tasks that appeared overwhelming. The OT followed up with these tasks by going over to his apartment and giving him additional support. The clinicians worked his service dog into their sessions as appropriate to give Mr. Doe an integrative experience and help him feel more at ease. Mr. Doe also had the opportunity to work with a Canine Companions facility dog in OT, PT movement group, and DMT.

Mr. Doe was given homework in all the disciplines, and he seemed to thrive with the practical hands-on tools that he could practice on his own and master over time. Whether it was specific physical exercises for PT, vision exercises for OT, articulation practice for SLP, deep breathing and reframing his negative thoughts for BH, or initiating a comic strip for AT, he frequently referenced using these tools outside of sessions, especially when feeling challenged. It appeared that this kind of practice motivated him and led to a sense of mastery while keeping him stimulated and challenged.

Mr. Doe benefited from being in a small cohort with three other veterans who had similar experiences and symptoms as he did. Having isolated himself and withdrawn from most activities, he began to make efforts to connect with his peers and increasingly expressed feeling safe and comfortable sharing in group settings. Active therapist-led (OT, SLP, AT) outings such as planning meals, visiting the grocery store, cooking together, and going to a bowling alley brought joy and invigoration to Mr. Doe as he saw himself doing things he had avoided for a long time. In addition, these group sessions provided opportunities for Mr. Doe to practice and reinforce the strategies he was learning in individual sessions. The individual sessions, meanwhile, prepared him to be ready for these outings; for example, in PT, he practiced strategies to decrease motion sensitivity; in OT and SLP, respectively, he developed and rehearsed visual and cognitive tools; and in BH, he learned to reframe negative thoughts and calm his nervous system through emotional regulation skills, such as breathing, grounding, and being present.

For three weeks, Mr. Doe engaged in a structured full-day program. He practiced and explored independently in the evenings and on the weekends. Nevertheless, one of the limitations of this approach is its brevity. Three weeks of new and integrative learning can certainly provide a reset by decreasing symptoms, supplying practical tools, bolstering confidence, and maybe most importantly, giving hope that change is possible and can be sustained. Nevertheless, after the three weeks, clients return to their homes where support is not always guaranteed. Varying levels of assistance from their local VA and health care systems, as well as family, environmental, or relational stressors that don’t necessarily resolve in three weeks, can complicate a smooth transition and impact the client’s positive trajectory. Consequently, the case manager and clinicians at MRIBH continue to communicate with the patients after their discharge and check in at set time points (e.g. 1-month, 6-months, and 1 year). Self-assessments (NSI, PCL-5 Short-Form, SIP-R, PART-O, Li-Sat 11, and PGIC) continue to be collected at these set time points. Monthly virtual MRIBH alumni sessions provide additional ongoing structure, comradery, and educational resources after program completion. With the groundwork laid, it is largely up to the clients to follow therapists’ recommendations and continue prioritizing their health and wellbeing.

## Conclusion

The treatment of chronic TBI in military veterans is complicated by symptoms of associated PTSD, depression, anxiety, and chronic pain, as well as unique environmental factors. A “one size fits all” approach is therefore not recommended. Instead, and as demonstrated in this case report, a highly individualized and collaborative interdisciplinary effort can be beneficial and can provide a springboard and positive trajectory for the veteran’s healing journey.

## Data Availability

The raw data supporting the conclusions of this article will be made available by the authors, without undue reservation.
